# Allergic Sensitization to Inhalant Allergens in the Upper Respiratory Tract—the B Cell Side

**DOI:** 10.1111/all.70229

**Published:** 2026-01-23

**Authors:** Ola Grimsholm, Mohammed Zghaebi, Bita Hambrecht, Tanja Kalic, Christopher C. Udoye, Rudolf Manz, Barbara Bohle, Katarzyna M. Sitnik, Julia Eckl‐Dorna, Heimo Breiteneder

**Affiliations:** ^1^ Department of Pathophysiology and Allergy Research, Center of Pathophysiology, Infectiology and Immunology Medical University of Vienna Vienna Austria; ^2^ Vienna Airway Lab, Department of Otorhinolaryngology, Head and Neck Surgery Medical University of Vienna Vienna Austria; ^3^ Department of Dermatology University Hospital St. Pölten, Karl Landsteiner University of Health Sciences St. Pölten Austria; ^4^ Institute for Systemic Inflammation Research University of Lübeck Lübeck Germany; ^5^ Department of Biomedical Sciences and Pathobiology University of Veterinary Medicine Vienna Vienna Austria

**Keywords:** allergen transport, B cells, epithelial barrier, IgE plasma cells, nasal mucosa, organoids

## Abstract

Allergic diseases are on the rise worldwide, driven by respiratory epithelial barrier dysfunction that promotes sensitization to inhalant allergens such as pollen, dust mites, pet dander, and fungal spores. These antigens trigger IgE‐mediated immune responses that lead to diseases such as allergic rhinitis (AR) and asthma. B cells play a central role by producing allergen‐specific IgE, presenting antigens, releasing cytokines, and forming memory B cells (MBCs). Their differentiation into IgE‐secreting plasma cells (PCs) mainly relies on T cell help, germinal center (GC) reactions, and/or extrafollicular responses and class switch recombination (CSR), which makes them important therapeutic targets. The nasal mucosa, as the first point of contact for allergens, acts both as a barrier and as an immunological site. In AR, IL‐13‐driven goblet cell hyperplasia and overproduction of mucus compromise the integrity of the barrier. Although the nasal microbiome can influence the immune response, its role in atopy remains unclear. Local B cell activity, including extrafollicular IgE production and ectopic GCs, enhances mucosal immunity. Epithelial cells detect allergens via pattern recognition receptors (PRRs) and release alarmins (IL‐25, IL‐33, TSLP), which can trigger type 2 inflammation. Proteases from allergens such as house dust mites (HDM) disrupt epithelial junctions, while pollutants, smoke, microplastics, and allergen‐derived metabolites further modulate immune activation. Allergens are transported to the lymph nodes by the passive flow to follicular dendritic cells (FDCs) or by active uptake by interferon regulatory factor (IRF) 4‐dependent conventional type 2 DCs, which activate T follicular helper (TFH) cells to drive IgE responses. Advanced lymphoid organoids that mimic the microenvironment of GCs offer promising models for the study of allergic sensitization but require improved standardization.

## 1 Introduction

Allergic diseases represent a constantly growing and considerable health burden worldwide. The extended epithelial barrier hypothesis proposes that damage to the epithelial barriers of the skin, respiratory tract, and digestive tract plays a decisive role in the increase in allergies [1]. Inhalant allergens are airborne antigens that trigger IgE‐mediated allergic reactions in susceptible individuals. These allergens mediate respiratory diseases such as AR and allergic asthma [2]. Epithelial barrier disruption of the respiratory mucosa contributes significantly to the pathogenesis of allergic diseases [3]. Sources for outdoor airborne allergens are mainly pollen grains of trees, grasses and weeds, and fungal spores [4]. Sources of indoor airborne allergens are predominantly dust mites, domestic pets, and fungal spores, but also cooking vapors of seafood [5, 6, 7, 8]. These allergens initiate immune responses at mucosal surfaces, leading to the activation of both innate and adaptive immunity.

B cells play a central role in the allergic cascade by producing allergen‐specific IgE antibodies that sensitize mast cells and basophils, enabling rapid degranulation upon allergen re‐exposure. IgE is generated not only systemically within secondary lymphoid organs but also locally at mucosal sites. Isolated localized production of allergen‐specific IgE can occur in the nasal mucosa and may give rise to nasal symptoms characteristic of local allergic rhinitis (LAR) [9]. This condition cannot be detected using conventional diagnostic approaches such as skin prick testing or measurement of serum allergen‐specific IgE. Instead, establishing a diagnosis of LAR requires a compatible clinical history followed by confirmation through a localized allergen provocation test. Beyond antibody production, B cells contribute to allergen‐specific immunity through antigen presentation, cytokine secretion, and the generation of MBCs [10, 11, 12, 13]. Type 2 MBCs expressing CD23 (low‐affinity Fc epsilon receptor) have recently been discovered to possess the IgE memory in allergy [12, 13]. Regulatory B cells secreting IL‐10 may also play a significant role in allergy through induction of tolerance during allergen immunotherapy (AIT) [14]. The differentiation of B cells into IgE‐secreting PCs is a tightly regulated process involving T cell help, the GC reaction, and CSR. However, in CD40 ligand‐deficient patients, IgE+ B‐cell responses have also been detected, indicating a potential T cell‐independent pathway [15]. Understanding the regulation and function of B cells in response to inhaled allergens is essential for unraveling the mechanisms of allergic disease and developing novel targeted immunotherapies.

Current research relies heavily on animal models and ex vivo systems, but these approaches have intrinsic limitations in replicating the complexity of the human immune environment, particularly epithelial–immune crosstalk [16]. Organoid‐based models, including those derived from tissue and peripheral blood mononuclear cells (PBMCs), are emerging as a promising platform to study human B‐cell responses and tissue‐specific immune interactions in a physiologically relevant context. Integrating organoid research into allergy and immunology not only bridges gaps left by traditional models but also holds great potential for personalized medicine and mechanistic discovery [17] (Box 1).

BOX 1 **Current knowledge and key recent advances.**

- The establishment of the epithelium of the respiratory tract and its innate receptors as a decisive factor in the development of the immune response to allergens.
- Identification of the role of environmentally polarized, migratory type 2 conventional dendritic cells (cDC2s) in the priming of T follicular helper cells, which can trigger IgE‐mediated allergic responses.
- Detection of tissue‐resident B cells and immature IgE‐secreting plasma cells in the nasal mucosa of patients with type 2‐mediated diseases.
- Detection of extrafollicular IgE production in human nasal polyps via a germinal center‐independent pathway involving local class switching of naïve B cells.
- Identification of a distinct subset of type 2‐polarized memory B cells, which hold the memory of allergen‐specific IgE.
- Establishment of human immune organoid models, including systems derived from tonsils and PBMCs, that successfully mimic key aspects of B cell activation, including affinity maturation processes.

## 2 The Nasal Mucosa—Site of Primary Contact

**FIGURE 1:**
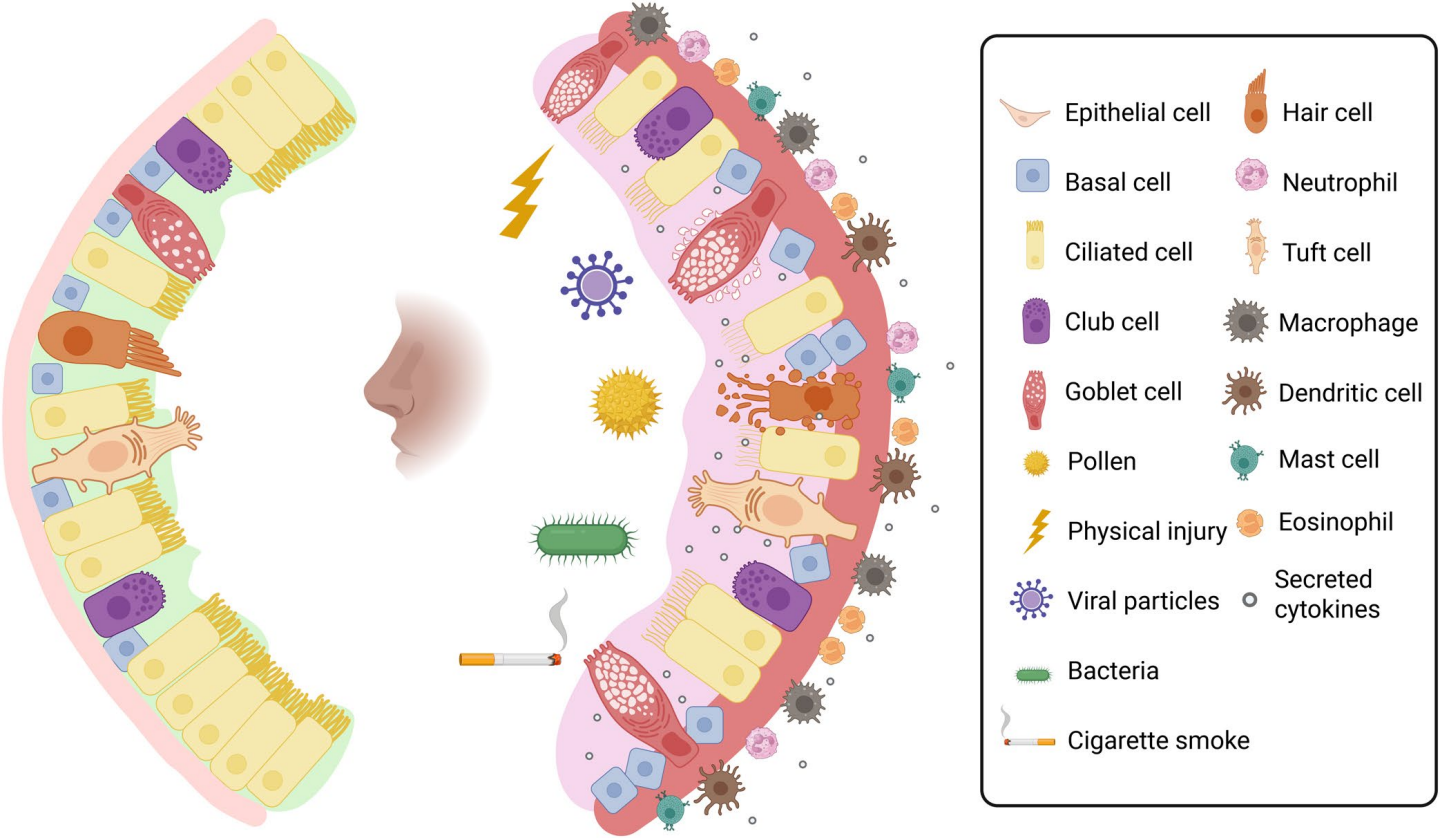
Nasal epithelium in normal and injured conditions. In the homeostatic and healthy nasal epithelium (left), ciliated cells form a motile but tight layer, and goblet cells are present in moderate numbers, with balanced mucin secretion resulting in a thin, clear mucus lining the apical surface. Upon epithelial disruption due to physical injury, viral infection, allergens, bacteria, or cigarette smoke (right), distinct changes occur that are characteristic of impaired barrier integrity. These include marked cilia loss and reduction in tight junctions, increased mucus secretion, and infiltration of immune cells. Colored annotations in the box on the right identify each cell type and pathological feature.

Although the surface area of the nasal cavity, at 150–200 cm^2^, is about 3500 times smaller than the epithelium covering the lower airways, it is often the first site of interaction with environmental stimuli. Though the cellular composition of the upper and lower respiratory tract exhibits some differences, they are tightly linked and often viewed as a single integrated organ system, consistent with the unified airway hypothesis [18]. The mucosa of the upper respiratory tract is a pseudostratified columnar ciliated epithelium composed of basal cells, ciliated cells, mucus‐secreting goblet cells, a few chemosensory cells, and—in the olfactory region—olfactory cells (Figure 1). Basal cells are important progenitor cells that can differentiate into specialized cell types. They are very diverse in healthy subjects, but their cellular diversity is greatly reduced in type 2‐mediated diseases [19]. Recent data confirm and extend these findings by demonstrating a shift of basal cells towards a diseased state characterized by an inflammatory signature and altered proliferation capacity in AR patients [20]. Goblet cells also play a key role in the disease, as they mediate one of the cardinal symptoms of AR through excessive mucus production. IL‐13 is known to trigger mucus production in both AR and asthma, but the involvement of neuropeptides in directly activating goblet cells via receptor interaction has also been discussed [21, 22]. Interestingly, the composition of mucus appears to differ between healthy controls and allergic individuals, with atopics having a greater number of apolipoproteins and imbalanced levels of cysteine proteases and antiproteases, which could affect the epithelial integrity and composition [23].

The nasal epithelium not only forms an important physical barrier (see below), but also a chemical and biological barrier that maintains immune homeostasis and separates immune cells from the commensal microbiota. The microbiome can affect B‐cell responses on several intersecting levels. Gut, lung, and nasal commensals produce short‐chain fatty acids (SCFAs: propionate, butyrate, acetate), which are well‐established inhibitors of histone deacetylase (HDAC) [24]. In particular, propionate and butyrate have been reported to reduce CSR to IgE and PC differentiation, both in mice and humans, in a dose‐dependent manner, although this effect was not specific to IgE alone [25]. Lower lung acetate levels also correlate directly with increased HDM‐specific IgE and type 2 inflammation [26]. On the other hand, dysbiosis can activate B cells indirectly through the “alarmin pathway” by altering the levels of pathogen‐associated molecular patterns (PAMPs) like LPS (a Toll‐like receptor (TLR) 4 ligand) and CpG DNA (TLR9 ligand). Through this route, apical stimulation of TLRs promotes tolerance [27], while TLR2 agonists have been reported to trigger epithelial cells to secrete TSLP and IL‐33 in human nasal polyps [28], thereby promoting type 2 immune responses. TSLP upregulates OX40L on DCs and polarizes naïve T cells to a Th2 phenotype that produces IL‐4 and IL‐13 and directs B cells to class switching to IgE [29]. Interestingly, *Staphylococcus aureus* can also act directly on B cells and surpass standard antigen presentation through the secretion of enterotoxins and Staphylococcal protein A (SpA), which plays the role of a B cell superantigen and directly amplifies polyclonal IgE production [30, 31].

Though the impact of selected microorganisms on IgE production has been elucidated, reports on the differential composition of the microbiome between AR and healthy controls are contradictory. Some studies report an increased microbial diversity in atopic patients, others indicate a decreased diversity, and yet others show no significant differences [32, 33, 34, 35]. Of note, specific members of the resident microbiota, such as *Streptococcus salivarius*, have been shown to enhance cytokine secretion by epithelial cells and to be associated with symptom aggravation [33]. Finally, recent large‐scale analyses demonstrate that temperature, humidity, surrounding greenery, and air pollutants such as NO_2_ and O_3_ shape nasal microbiome diversity and are associated with respiratory health outcomes [36].

Once the allergen has crossed the epithelial barrier, it comes into contact with a variety of immune cells located in the connective tissue of the nasal mucosa [37]. DCs are among the first to come in contact with the allergen and can even extend their processes into the airway lumen for antigen sampling (see also section below) [38]. In previously sensitized patients, allergens bind to receptor‐bound IgE, either on effector cells leading to their activation, or on antigen‐presenting cells such as DCs or B cells, facilitating allergen presentation to T cells [39]. The biology of tissue‐resident B cells has been fundamentally revised in recent years. For example, intravenous labeling and parabiosis techniques have shown that the lung, liver, kidney, and urinary bladder have a notable proportion of permanently resident B cells [40]. In the nasal mucosa, activated IgD+ naïve‐like intermediate B cells were reported as the primary source of mucosal IgE antibody‐secreting cells (see section below) [41]. These cells are formed extrafollicularly and independently of classical GC reactions. However, this does not exclude that GCs contribute to local tissue immunity, as GCs were shown to persist in the upper airways even under steady‐state conditions [42]. Ectopic GCs have also been identified within the nasal turbinates themselves in nasal swabs from healthy volunteers and reported to contribute to increased antibody production [43]. Recent findings also indicate an accumulation of MBCs and the presence of immature IgE‐secreting cells in the nasal mucosa of patients with polyps, another type 2 immunity‐driven disease [44, 45]. The nasal mucosa is a key immunological barrier where epithelial changes and microbial products, like SCFAs, directly impact B‐cell function, inhibiting IgE class switching. Advances include defining tissue‐resident B cells as local IgE sources. However, unresolved questions remain regarding the specific role of nasal dysbiosis and how these localized B‐cell responses contribute to type 2 diseases.

## 3 Interaction of Inhalant Allergens and Epithelial Barriers—The Role of Pattern Recognition Receptors, Barrier Integrity, and Exogenous Modulators

The airway epithelium plays an active role in immune responses to allergens [46]. Upon interaction with allergens, epithelial cells release alarmins, which lead to activation of ILC2s and Th2 differentiation [47]. During the development of a type 2 immune response, an innate response originates from epithelial cells via the alarmins TSLP, IL‐25, or IL‐33, which induce type 2 innate lymphoid cells (ILC2s) to release type 2 cytokines in the early phase of allergic sensitization [29]. This provides initial IL‐4 and IL‐13 for B‐cell activation and IgE class switching before full Th2 cell differentiation, especially in the extrafollicular pathway, which quickly produces short‐lived antibody‐secreting cells [48]. Epithelial cells detect inhaled allergens mainly via PRRs or through damage caused by allergenic proteases [49, 50, 51]. The HDM allergen Der p 2, for instance, binds to TLR4, leading to a release of IL‐25, IL‐33, TSLP, and GM‐CSF [52]. This interaction is based on the structural homology between Der p 2 and myeloid differentiation factor 2 (MD‐2), a coreceptor for TLR4. Extracts from various pollen sources and from cat dander were also shown to interact with MD‐2, leading to MD‐2‐dependent NF‐κB activation, CXCL8 secretion, allergic sensitization, and airway inflammation, but the purified allergens were not tested [53]. A study in a mouse model of birch pollen allergy identified NLRP3 in an inflammasome‐independent role as an important driver of birch pollen‐induced allergic immune responses [54].

Allergens and extracts from allergen sources can damage the epithelial barrier and trigger specific downstream responses. Exposure of nasal epithelial cells to the HDM cysteine protease Der p 1 resulted in cleavage of the tight junction protein claudin‐1, reduction of transepithelial electrical resistance (TEER), and secretion of IL‐6 in patients with HDM‐induced AR, but not in control subjects [55]. HDM also triggers the extracellular release of damage‐associated molecular patterns, including ATP, which is recognized by G‐protein‐coupled receptors such as P2Y2 on airway epithelial cells, leading to IL‐33‐driven type 2 inflammation [56]. Exposure of the airway epithelium to allergenic proteases from papaya, *Aspergillus oryzae*, and HDM led to the formation of stress granules, cleavage of gasdermin D, and release of IL‐33 into the extracellular space [57]. The ripoptosome is an approximately 2 MDa cell death‐inducing signaling platform that contains the core components RIP1 (receptor‐interacting protein kinase 1), FADD (Fas‐associated death domain protein), and caspase 8 and is negatively regulated by proteins such as cFLIP (cellular FLICE‐inhibitory protein; FLICE = FADD‐like IL‐1β‐converting enzyme) [58]. The stoichiometric composition of the ripoptosome determines the cell fate, i.e., apoptosis versus necroptosis, or cell survival [59]. A diverse group of animal and fungal but not plant allergen sources induced the intracellular production of mature IL‐33 concurrently with RIPK1 phosphorylation and caspase 8 activation [60]. This suggested that allergen stimulation of epithelial cells activated the ripoptosome, leading to mature IL‐33 production independently of cell death.

Many allergens bind small molecular ligands, and allergen sources contain various compounds in their matrix, including lipids, carbohydrates, and other metabolites, which may modulate cellular responses during the allergic sensitization [61, 62, 63]. In contrast to the effect of barrier‐disrupting external proteases, low molecular weight components (LMCs), smaller than 3 kDa, have been shown to have the opposite biological effect. LMCs derived from various pollen sources increased the TEER of bronchial epithelial cell monolayers in vitro, accompanied by the polarized, apical release of GM‐CSF [64]. Another study on birch pollen LMCs demonstrated their similar, barrier‐enhancing properties in bronchial epithelial cells, accompanied by increased release of IL‐6 and decreased release of CCL5 and TNFα [65]. The impact of LMCs on bronchial epithelial cell barriers was also shown for fish, which is able to sensitize not only by ingestion but also by inhalation and skin contact [66].

Transcriptome profiling of airway epithelial cells upon exposure to inhalant allergens and other environmental factors is a useful tool to identify immune processes. Using primary human bronchial epithelial cells from donors with fatal asthma and non‐asthmatic healthy donors, and exposure to HDM and LPS, a stronger reaction of the cells upon exposure to the combination of these two stimuli was shown [67]. Cells of asthmatic donors also reacted with a greater number of differentially expressed genes (DEGs) than cells of non‐asthmatics [67]. Sudharson et al. investigated transcriptomic profiles of nasal epithelial cells following nasal provocation with birch pollen extract among participants with or without birch pollen allergy [68]. A higher number of DEGs and canonical pathways related to immune cell recruitment and proinflammatory cytokine signaling among allergic participants were observed [68].

Factors other than allergen sources contribute to shaping the immune responses during exposure to allergens [69]. Dysbiosis of the nasal microbiota, which is linked to chronic inflammatory disorders, including AR and chronic rhinosinusitis [70], influences B‐cell responses via various mechanisms. Bacterial fermentation produces SCFAs that fuel the metabolic activity of B cells, act as HDAC inhibitors, thus influencing gene expression to support B‐cell differentiation, and promote regulatory B cell (Breg) development [71]. Dysbiosis leads to a decrease in SCFA‐producing species and an increase in opportunistic or pathogenic bacteria in the respiratory tract, resulting in lower SCFA levels [72]. Microbial metabolites also significantly impact epithelial cytokine release, with SCFAs often promoting an anti‐inflammatory state and indole derivatives maintaining epithelial barrier function [73]. TLRs recognize conserved components of microorganisms and are expressed on human nasal epithelial cells, B cells, and DCs. Dysbiosis of the microbiota influences B‐cell responses by altering the signals that DCs receive via TLRs, thereby shaping the T‐cell help that is critical for B‐cell maturation and function [74]. The nasal cavity is a major reservoir for opportunistic pathogens, including *Staphylococcus aureus* , which contributes to dysbiosis in allergic nasal mucus [75]. The *S. aureus* protein A (SpA) is a B‐cell superantigen that binds to the B‐cell receptor (BCR) [76] and has complex effects on B cells, including inducing proliferation and apoptosis, promoting polyclonal expansion, and disrupting long‐lived PC formation [77].

Lee et al. investigated the composition and functional genes of the airway microbiome from young adults, elderly asthma patients, and non‐asthmatic subjects [78] A higher expression of genes related to lysine degradation, N‐glycan biosynthesis, caprolactam degradation, and the PPAR signaling pathway was detected in the airway microbiota of non‐asthmatics than of asthmatics [78]. Such genes may play a role in the reduction of inflammation, indicating an important role of the airway microbiome in immune responses. Currently, the most significant knowledge gap is the paucity of specific data on the production and function of key microbial metabolites, such as SCFAs, directly within the nasal cavity. Future research will need to characterize the unique metabolic landscape of the nasal microbiome.

Exposure to environmental factors, especially the countless new chemicals that have been introduced into modern life, plays a major role in the development of allergic diseases [79]. In particular, cigarette smoke [80, 81], household detergents [82], microplastics [83], and air pollutants such as diesel exhaust particles (DEP) [84, 85] actively promote allergic sensitization through their disruptive influence on epithelial barriers and subsequent modulation of adaptive immunity [86]. Cigarette smoke and vaping are significantly associated with several changes in B‐cell function, including increased class switching, clonal expansion, and a shift towards IgA‐producing cell populations [87]. Household detergents can influence B cells indirectly, primarily by affecting epithelial cells and triggering inflammatory responses, as well as potentially directly affecting cell membranes [82]. Microplastics can potentially act as adjuvants by activating the NLRP3 inflammasome, an important component of the innate immune response, in accessory cells such as macrophages and DCs, which can then influence adaptive immunity [88]. DEP mediates the up‐regulation of IL‐33, IL‐25, and TSLP and a Th2 activation via the aryl hydrocarbon receptor (AhR), a ligand‐activated transcription factor, thus linking environmental pollution and severe asthma [85]. In BALB/c mice, DEPs induced local B‐cell IgE class switching that took place in preexisting inducible bronchus‐associated lymphoid tissues of epithelial barrier disruption to subsequent B‐cell activation and IgE responses need to be further critically analyzed. The B‐cell responses underlying allergic sensitization are the result of a complex interplay of epithelial barrier dysfunctions, the ensuing cascades of molecular and cellular signals, as well as microbiome signals and the biological activities of the allergens or components of the allergen source. In conclusion, key advances reveal the epithelium actively initiates Type 2 immunity by releasing alarmins upon allergen detection or damage, quickly driving B‐cell IgE class switching. The unresolved question is the specific, localized influence of microbial metabolites (SCFAs) and of environmental pollutants on B‐cell differentiation and IgE production, highlighting a critical knowledge gap in allergic sensitization.

## 4 Transport of Allergens into the Lymph Node

Two distinct mechanisms enable the transport of allergens into the lymph nodes (LNs): passive transport through the flowing lymph and active transport by tissue migratory DCs.

### 4.1 Passive Allergen Transport via the Lymph

**FIGURE 2:**
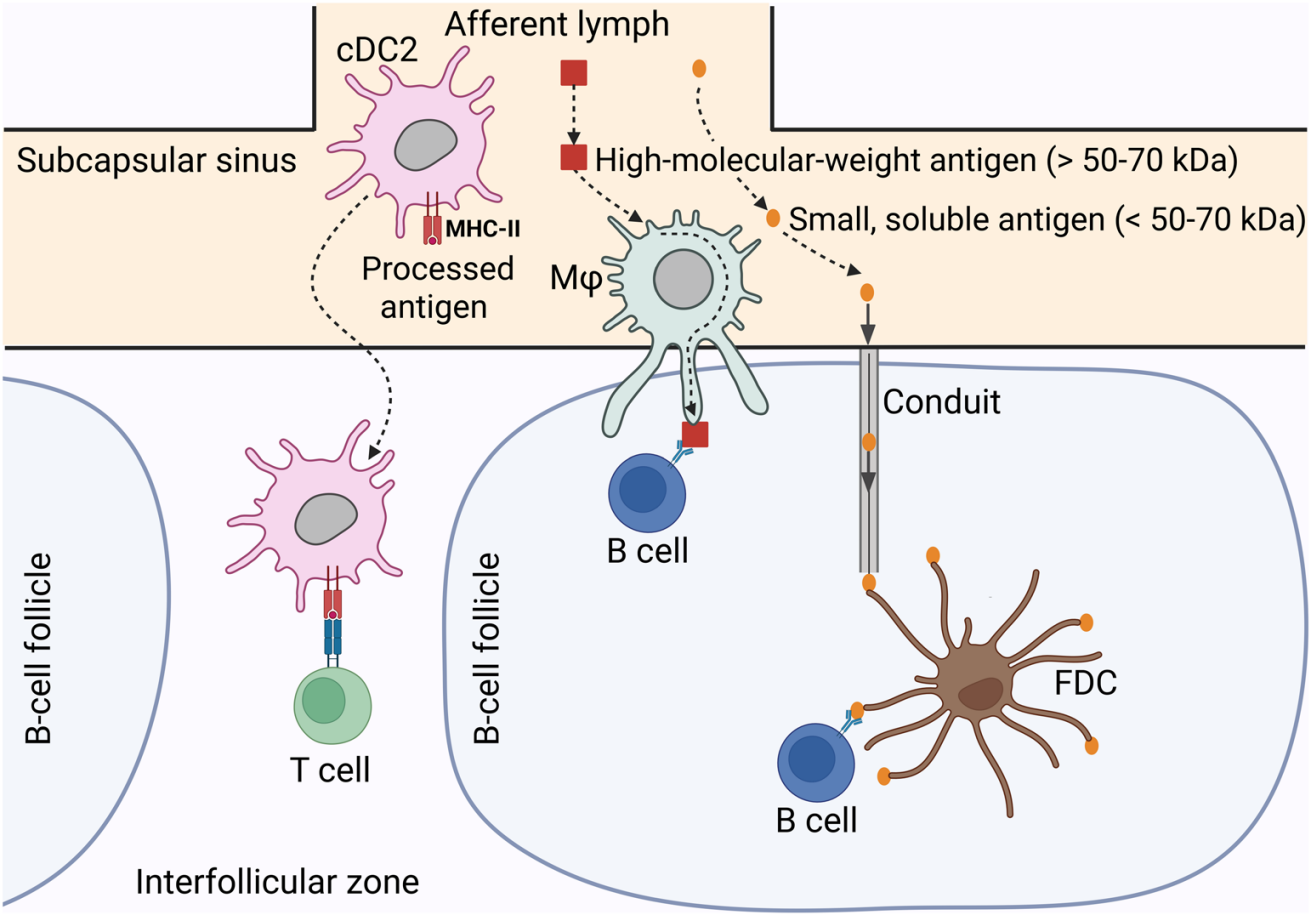
Transport of allergens into the lymph node. B cells become activated by native allergens passively carried by the lymph into the LN subcapsular sinus (SCS). Antigen transfer from the SCS into the B‐cell follicle is possible via two routes. (1) Small, soluble antigens (< 50–70 kDa) gain access to the conduit system, through which they are channeled deep into the follicle and deposited on FDCs. (2) High‐molecular‐weight antigens (> 50–70 kDa) are picked up and shuttled across the SCS floor by subcapsular Mf. In addition, migratory cDC2s bring processed antigens into the LN and present them to naïve T cells in the interfollicular zone, leading to the priming of TFH cells that promote the generation of IgE B‐cell responses.

Upon reaching the LN via afferent lymphatic vessels, lymph is discharged into a superficial, narrow space below the LN capsule, the subcapsular sinus (SCS). Conveyed by the SCS, the lymph flows around the lymphocyte‐containing paracortex before draining into the medullary sinuses and exiting the LN via efferent lymphatic vessels (Figure 2). Early research into the routes of antigen access to the paracortex suggested a mechanism involving B‐cell acquisition of unprocessed, native antigens from the surface of T‐zone DCs [89]. However, the current consensus is that antigens in the lymph are acquired by antigen‐specific naïve B cells in the underlying follicles and not in the T‐zone and that this process does not require B cell migration through the T cell area or exposure to DCs [90, 91, 92]. Whether or not the antigenic content of the lymph may enter the B‐zones of the paracortex depends on the size of the antigen. Experiments tracking injected labeled antigens by microscopy have shown that soluble antigens do not percolate freely from the SCS through the follicles [93]. Instead, low‐molecular‐weight antigens with a molecular mass of less than 50–70 kDa (this category includes many clinically relevant allergens such as Bet v 1, Der p 1, Der p 2, Fel d 1) percolate from the SCS into the follicle core via a specialized transport system composed of enclosed conduit tubes with a collagen fiber core [90, 91, 94]. The conduit system physically connects with and relays antigens to the network of FDCs [90], a population of fibroblasts located in the follicle center that specializes in the long‐term display of native, undegraded antigens to B cells [95]. In contrast to small antigens, molecules with a high molecular weight (> 50–70 kDa) cannot enter the conduits but are carried across the SCS wall by macrophages residing in the SCS in areas adjacent to the B‐cell follicles [96]. In mice immunized with high‐molecular‐weight antigen–antibody complexes, the antigen displayed on the surface of SCS phagocytes serves to activate cognate B cells both directly and following its deposition onto FDCs by non‐cognate B cells [96]. It remains unknown which, if any, of the presently known routes of antigen transport to the lymph nodes are relevant for allergic sensitization of B cells.

### 4.2 Active Allergen Transport by Tissue Migratory Dendritic Cells

Conventional DCs (cDCs) continuously sample soluble and particulate tissue contents and, via lymph vessels, carry them into the draining LNs, where they prime T cell responses by presenting processed antigenic peptides in the context of major histocompatibility complex (MHC) I and II molecules to naïve CD4 and CD8 T cells [97]. While cDC maturation under homeostatic conditions typically leads to T cell tolerance, cDCs acquire the capacity for immunogenic T‐cell activation following contact with pathogens or, as in the context of allergic sensitization, through signals released upon allergen‐induced barrier tissue damage [98, 99]. Although B‐cell priming does not rely on antigens brought into the LNs by migratory DCs but rather is initiated by antigens delivered passively via the lymph, active antigen transport by cDCs has an important role in the generation of IgE in responses to allergens via inducing TFH cells (see Ref [100] for an excellent review on the topic). Notably, the priming of TFH cells following intranasal and subcutaneous immunization of mice (including that with an allergenic protease) specifically depends on the activation of naïve CD4 T cells by an IRF‐4 dependent, ontogenically distinct subset of cDCs, termed cDC2 [101, 102]. Interestingly, due to their specific ability to sense the B zone‐specific chemokine CXCL13 via CCR5 and their limited responsiveness to chemokines expressed in the T zone, migratory cDC2 arriving in the LN uniquely carry antigens into the T‐B border of the LN, where TFH cell priming occurs [102, 103, 104] (Figure 2). The key advance is the distinction between two allergen transport routes into the lymph node: the passive delivery of small molecules via conduits to B‐cell follicles and the active transport by migratory cDC2s for TFH priming. One unresolved question is identifying which pathway governs allergic sensitization and how DC‐delivered antigens integrate with B‐cell activation to drive IgE responses.

## 5 Activation of Naïve B Cells

Naïve B cells co‐express IgM and IgD and make up the initial repertoire that will encounter antigens in peripheral tissues. In the case of allergies, their activation is the first step towards the generation of allergen‐specific IgE. Loading the FcεRI receptors with allergen‐specific IgE permits subsequent triggering of an allergic response. Subsequent allergen exposure triggers the degranulation of mast cells and basophils via this receptor. Mature naïve B cells have historically been divided into three primary populations in mice: B1, follicular (FO) B2, and marginal zone (MZ) B cells. This division remains controversial in human immunology. Over the years, only limited attention has been given to the investigation of the activation of naïve B cells during IgE‐mediated allergies, albeit their fundamental importance for the pathological mechanisms.

The main insights into the mechanisms underlying the activation of naïve B cells in IgE‐mediated allergies so far come from mouse studies, where one study by Sallusto and colleagues demonstrated that naïve B cells can pick up allergen independent of their BCR, process it, and present it to naïve T cells [10]. Innate‐like B1 cells have recently been viewed as important in the early stages of allergy in mouse models. B1 cells can express and secrete natural antibodies (NAbs) in the form of germline sequences, primarily of the IgM isotype, and they show broad cross‐reactivity with conserved molecular patterns [105]. These NAbs can recognize damage‐associated molecular pattern molecules (DAMPs) and serve as an alert to mount an immune response. In models of allergic airway disease, B1 cell‐derived NAbs initiate a type 2 immune response. This response facilitates subsequent interactions between Th2 cells and naïve B cells, ultimately driving the differentiation into IgE‐producing B2 cells [105]. By binding to DAMPs, these NAbs contribute to early cytokine signal initiation (i.e., IL‐4, IL‐5), which correlates with the Th2 inflammatory profile and adaptive development phase that characterizes all allergic responses. This suggests that the innate‐like B cells can also contribute to IgE production by mechanisms that are different from the high‐affinity response that occurs later in the allergic immune response. On the other hand, FO B2 cells' ability to activate and polarize T cells to Th2 cells is dependent on their expression of major histocompatibility complex (MHC) class II molecules and costimulatory proteins such as CD23 and CD40, and they were reported to have higher levels of expression of these molecules in allergic settings, further enhancing T cell proliferation and cytokine production to specific allergens [106].

**FIGURE 3:**
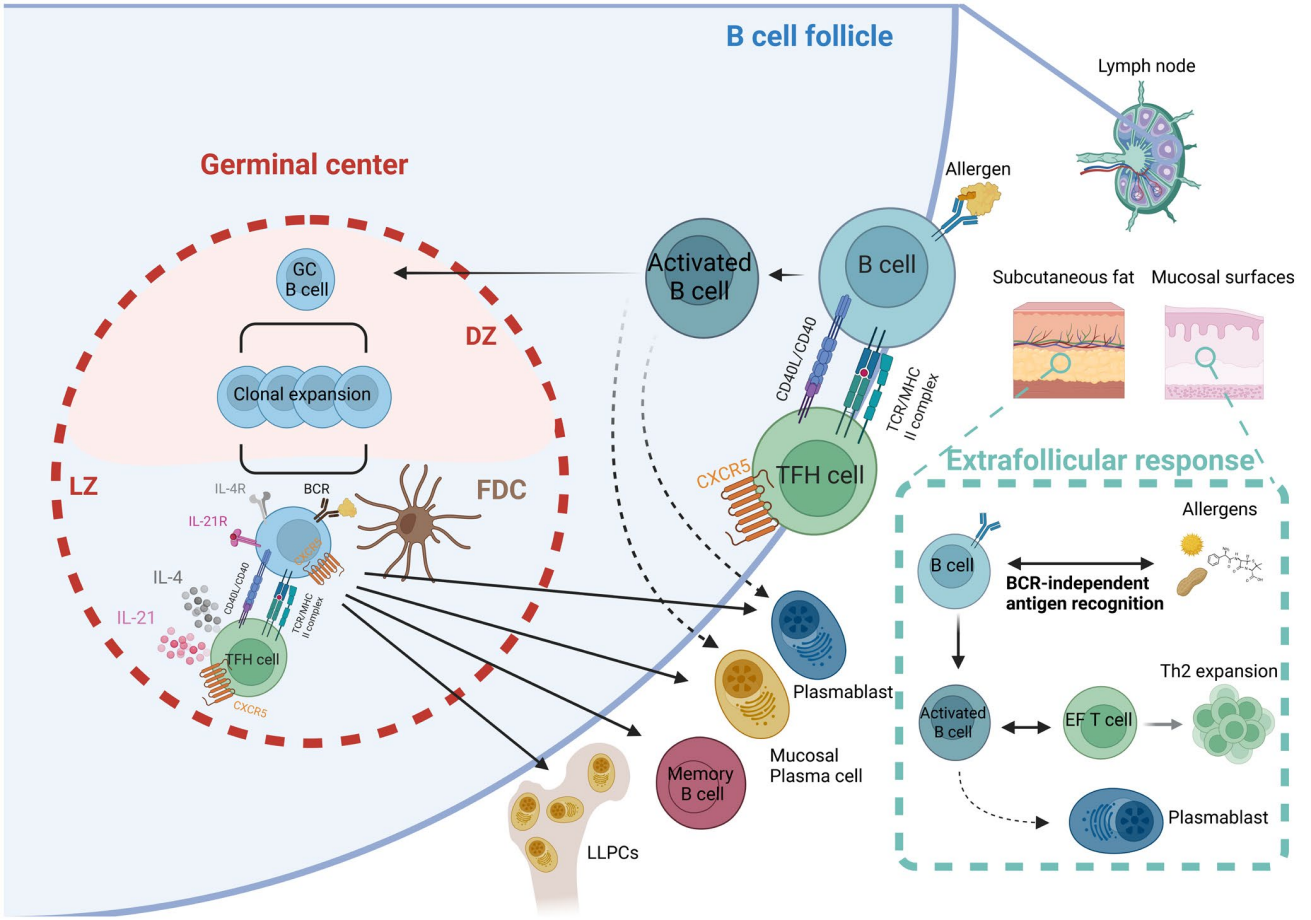
Pathways leading to IgE switched B cells in IgE‐mediated allergy: Germinal center reaction and extrafollicular response. The two models, germinal center (GC; to the left) and extrafollicular (EF; to the right), depict the major events leading up to plasma cells/plasmablasts and/or memory B cells from each pathway. Some key molecular factors are indicated, such as IL‐4 and IL‐21. Abbreviations: BCR, B‐cell receptor; DZ, dark zone; EF, extrafollicular; FDC, follicular dendritic cell; GC, germinal center; LLPC, long‐lived plasma cell; LZ, light zone; TFH, T follicular helper cell.

Two recent studies have investigated the activation of the local B cell responses in inflamed lungs in mice. Wu and colleagues reported that IgE switching predominantly occurs locally, in this case of inhaled allergen in the lung, from mainly IgG1 MBC precursors [107]. However, the origin of those IgG1 MBCs was unclear from their study. They further showed that TH2 cells producing IL‐4 in the lung might be the driving factor, and they speculated that the lung might be a more permissive environment for IgE switching due to the lack of true germinal centers. It has also previously been demonstrated that IL‐21 (most often produced by TFH cells) can inhibit switching to IgE at lower concentrations of anti‐CD40, possibly translating to environments with less T‐cell help [108]. Furthermore, in a study on human nasal mucosa, it was shown that extrafollicular IgD+ naïve‐like cells were rather the cells of origin for IgE antibody‐secreting cells [41]. They demonstrated that a GC‐independent pathway to IgE‐secreting PCs also exists in the nasal mucosa, originating from an antigen‐activated IgD+ B cell (likely a naïve B cell). Thus, it seems likely that there are two possibly parallel pathways of activation of naïve B cells leading to IgE‐switching, where one comes directly from IgD+ naïve‐like cells and the other one via IgG1 intermediate, although the latter one still has to be tracked down to its origin (Figure 3).

Recent experimental models have begun to explore the therapeutic potential of directly modulating naïve B cell activation to induce tolerance rather than hypersensitivity. Notably, adoptive transfer experiments have demonstrated that isolating and transferring allergen‐expressing B cells into naïve recipients, when combined with immunomodulatory preconditioning using agents such as rapamycin and anti‐CD40L antibodies, can establish long‐term, antigen‐specific tolerance, thereby preventing the development of IgE‐mediated allergic responses [109]. In addition, The TLR 5 ligand—flagellin A—has been used to construct a fusion protein composed of recombinant flagellin (rFla) in addition to the major birch pollen allergen Bet v 1 (rFlaA:Betv1) that is capable of inducing a subset of IgA+ regulatory B cells (Bregs) from naïve B cells, and thereby suppressing Th2 inflammation [110]. The transient administration of a B cell maturation antigen x CD3 (BCMAxCD3) bispecific antibody as a strategy to deplete allergen‐specific IgE has been explored [111]. These studies provide a promising proof‐of‐concept that manipulating the early activation stages of B cells and their subsequent differentiation trajectories can reshape the immune response from a pathogenic, allergy‐prone state to one of long‐lasting tolerance.

## 6 IgE Plasma Cell Formation and Antibody Responses to Allergens

### 6.1 IgE Plasma Cells

While most IgE is formed within GCs, where Ig class switching to IgE mainly occurs via sequential switching via another Ig subclass, IgE can also be formed by direct Ig class switching in extrafollicular reactions [112, 113] (Figure 3). Within GCs, where affinity maturation also occurs, formation of IgE and IgG PCs follows distinct kinetic patterns. In both mice and humans, IgG1 B cells typically remain in GCs for extended periods of days to weeks. In contrast, IgE B cells follow an accelerated differentiation pathway where they rapidly upregulate the PC master regulator Blimp‐1 (encoded by *prdm1*) and exit the GC as plasmablasts or (mainly) short‐lived PCs significantly faster than their IgG counterparts [114, 115]. Studies using IgE‐reporter mice and lineage tracing have revealed that IgE B cells appear in GCs only transiently and at low frequency [116]. They are predominantly localized in the dark zone of the GC and are notably scarce in the light zone, where B cells typically interact with TFH cells and undergo selection. This spatial restriction results from IgE‐switched GC B cells downregulating CXCR5, the receptor for the follicular chemoattractant CXCL13, thus impairing their migration into light zone areas. On this note, it was later more elaborately shown by C. Allen and colleagues that IgE B cells receive reduced T cell help and antigen presentation in the GC, accelerating their exit [117]. Furthermore, transcriptional analysis of human IgE‐expressing cells demonstrates that IgE^+^ B cells exhibit a transcriptional profile characterized by high IRF4 and Blimp‐1 levels and low Pax5 and Bcl‐6 expression, predisposing them to rapid PC differentiation [118]. This rapid differentiation of IgE+ cells is evidenced by the disproportionately high frequency of IgE‐secreting cells among IgE‐switched cells shortly after sensitization, compared to IgG1‐switched cells [115, 119]. IgE PCs also possess a unique transcriptional signature that distinguishes them from PCs that produce other antibody isotypes. Research by Orengo and colleagues using an HDM allergy model revealed that IgE PCs differentially express CD19, BAFF‐R, TACI, CD138, CD98, and Irf4 compared to other PCs [120]. The preference of IgE+ B cells for becoming PCs rather than MBCs raised the question of how IgE memory is maintained in allergic individuals. Though IgE+ MBCs seem to be elevated in the peripheral blood of children with food allergy and allergic asthma, their overall frequencies are exceedingly rare [121, 122]. Recent studies have now identified a unique somatically mutated MBC2 population in humans and mice, which mainly express IgG1 and IgG4 antibodies, which upon reactivation show a strong tendency for IgE production [12, 13], Moreover, their frequencies are increased in atopic disease and correlate with serum IgE levels in allergic individuals [13].

Of note, MBC2s contain some IgE+ cells, which however seem not to correlate with allergic disease. Although these findings support the idea that IgG+ MBCs resemble allergenic IgE+ PC precursors, signaling via the IgE‐BCR was essential for mounting serum IgE memory in a transgenic mouse model [123]. IgE BCR expression on murine B cells was found to be low, but high on IgE+ PCs in an acute mouse model [124]. On the other hand, very recent data from two different labs using mouse models of IgE‐mediated allergy demonstrate that the spleen is an important reservoir for long‐lived IgE+ PCs. These PCs have a similar transcriptional profile as PCs expressing other isotypes but a higher expression of some survival genes encoding BCMA and TACI [125, 126]. However, no data are so far available from human spleen of allergic donors that could confirm these results. A subset of long‐lived murine IgE PCs homes to the bone marrow and sustains pathogenic IgE production, driving anaphylaxis [127]. On the other hand, we know that human bone marrow is at most a minor reservoir of IgE expressing PCs [120].

### 6.2 Allergen‐Specific Antibody Responses

Allergen‐specific IgG and IgA antibodies in humans are generally considered to be beneficial for allergic patients. Their levels rise as a result of the regular administration of allergens to an allergic individual during AIT. A fraction of these antibodies inhibits the interaction of IgE and allergen, thereby preventing crosslinking of IgE‐loaded FcεRI molecules on effector cells [128]. In addition, these antibodies reduce allergen uptake by B lymphocytes via the low‐affinity IgE receptor CD23. Up to now, the so‐called IgE‐blocking activity has been attributed mostly to IgG4. Recent studies demonstrated the ability of allergen‐specific IgA and IgG1 induced by subcutaneous and sublingual immunotherapy (SCIT and SLIT) to act as blocking antibodies [129, 130, 131]. Moreover, increased allergen‐specific IgG2 levels were reported with SLIT, but the blocking effect of these antibodies was not investigated [132].

But what characteristics make an allergen‐specific antibody an IgE‐blocking antibody? So far, the inhibitory activity of mAbs could be positively correlated with the quantity and affinity for the allergen [133]. While earlier work with mAbs of identical specificity but different subclasses found a comparable blocking activity [134], more recent studies reported a biphasic response of IgE‐blocking antibodies in AIT with an initially dominant protective role of allergen‐specific IgG1 followed by IgG4 [131]. Several studies indicated that functional blocking requires binding to sites mostly overlapping with IgE‐epitopes [135, 136, 137]. This was recently further supported by epitope‐resolved immunoassays providing evidence for a clonal relationship between pre‐AIT existing IgE and therapy‐induced IgG [138]. Nevertheless, more than one IgE‐epitope on an allergen needs to be blocked by high‐affinity IgG for a successful reduction of clinical symptoms, as demonstrated by passive immunotherapy with mixtures of at least two mAbs specific for non‐overlapping epitopes [139, 140]. The control of the generation of blocking versus non‐blocking IgG during allergic immune responses is not well understood. Extrafollicular IgE derived by direct class switch from IgM progenitors is seen in patients and mouse models [41, 113], and due to its potentially different fine specificity and epitope binding, it could potentially escape blocking by IgA and IgG antibodies. However, low‐affinity IgE poorly stimulates mast cells, and only GC‐derived high‐affinity IgE, not low‐affinity extrafollicular IgE, can cause anaphylaxis [141, 142]. Therefore, GC‐derived and affinity‐matured IgE appear to be the most relevant for severe allergic reactions. Accordingly, unpublished data from Manz and colleagues suggest that introducing divergent hypermutations in IgE clones, as compared to clonally related IgG, is associated with the development of allergic anaphylaxis in a murine food allergy model. These mutations alter IgE allergen‐binding properties within the GC, which promotes avoidance of IgG‐mediated blockade, and similarly, mutated “divergent IgE” was also seen in patient samples [143]. Hence, despite the fact that most IgE clones are deleted within GC, some “divergent” IgE clones with altered antigen‐binding sites compared to blocking IgG antibodies seem to escape. The precise nature of the changes of divergent IgE‐allergen‐binding remains unclear, but potentially affects affinity, epitope recognition and diversity, and/or antibody flexibility [144]. Competition among individual B‐cell clones for stimulation by antigen and TFH cells is a driving force behind clonal selection within the GC [145], and the development of high‐affinity, highly allergenic IgE requires help from TFH 13 cells [142]. Together, these findings may indicate that the introduction of new allergen binding properties within the GC could help “divergent IgE” clones escape competition from IgG clones, allowing for efficient help from TFH13 cells. Suppression of high‐affinity IgE through inhibition of TFH13 cells or boosting the generation of IgA and IgG with similar allergen‐binding properties to the allergenic IgE antibodies may represent novel strategies to improve AIT further. Such new therapies may also take into account that IgE+ PCs express high levels of IgE as BCR on their surface, with crosslinking of this receptor possibly suitable to induce apoptosis of pre‐existing IgE+ PCs [124].

### 6.3 B Cell‐Mediated Immunoregulation

Because of their unique ability to produce antibodies, the importance of other key B cell functions for developing and regulating allergic diseases may be overlooked. During chronic immune stimulation, B cells are potent antigen‐presenting cells and modulate antigen‐specific T cell responses. In the context of an allergic response, CD23+ B cells could capture IgE–allergen complexes to increase the specific T cell response, a mechanism that potentially contributes to epitope spreading [146]. In addition, murine and human B cells and PCs can produce a variety of cytokines. IL‐10 production by Bregs and PCs was shown to have considerable effects on the outcome of T cell activation and innate effector cell function in a variety of murine models [147, 148]. In‐depth characterization of allergen‐specific B cells in patients allergic to cow's milk indicates that AIT and remission are associated with a shift from relatively high IgE and IgG1 to very little IgG1 and high IgG4 expression, while IgE was merely unaffected. In addition, allergen‐specific B cells showed upregulation of genes associated with regulatory B cells [149]. These findings confirm that AIT alters the relative ratios of allergenic versus protective antibodies; in addition, they provide evidence for induction of functional changes within the B cells towards a tolerogenic regulatory B cell phenotype. However, multiple subsets of regulatory B cells, plasmablasts, and PCs exist [147, 148], and both the identity and precise role of these regulatory B cell subsets in allergen‐tolerance remains to be elucidated.

## 7 Organoids—A Model System to Study B‐Cell Activation

Organoids are self‐organizing, multicellular structures grown in vitro from stem cells, tissue‐derived progenitors, or primary cells such as PBMCs. Organoid models of lymphoid tissues are valuable tools to recreate complex immune environments by preserving cell heterogeneity and mimicking the architecture and functionality of their tissue of origin, making them uniquely suited for studying B cells.

B cells play a central role in allergic sensitization through antigen uptake, activation, antibody class switching to IgE, and differentiation into MBCs and PCs. These processes are tightly regulated by interactions with T helper cells and the surrounding stromal and epithelial microenvironment, especially in secondary lymphoid organs like the mucosa‐associated lymphoid tissue (MALT) [150]. However, mimicking these sequential and spatially organized activation steps in vitro is still challenging. Organoid models now offer a solution as they potentially allow the controlled recreation of GC‐like reactions and antigen‐specific B cell activation in human‐derived systems.

**FIGURE 4:**
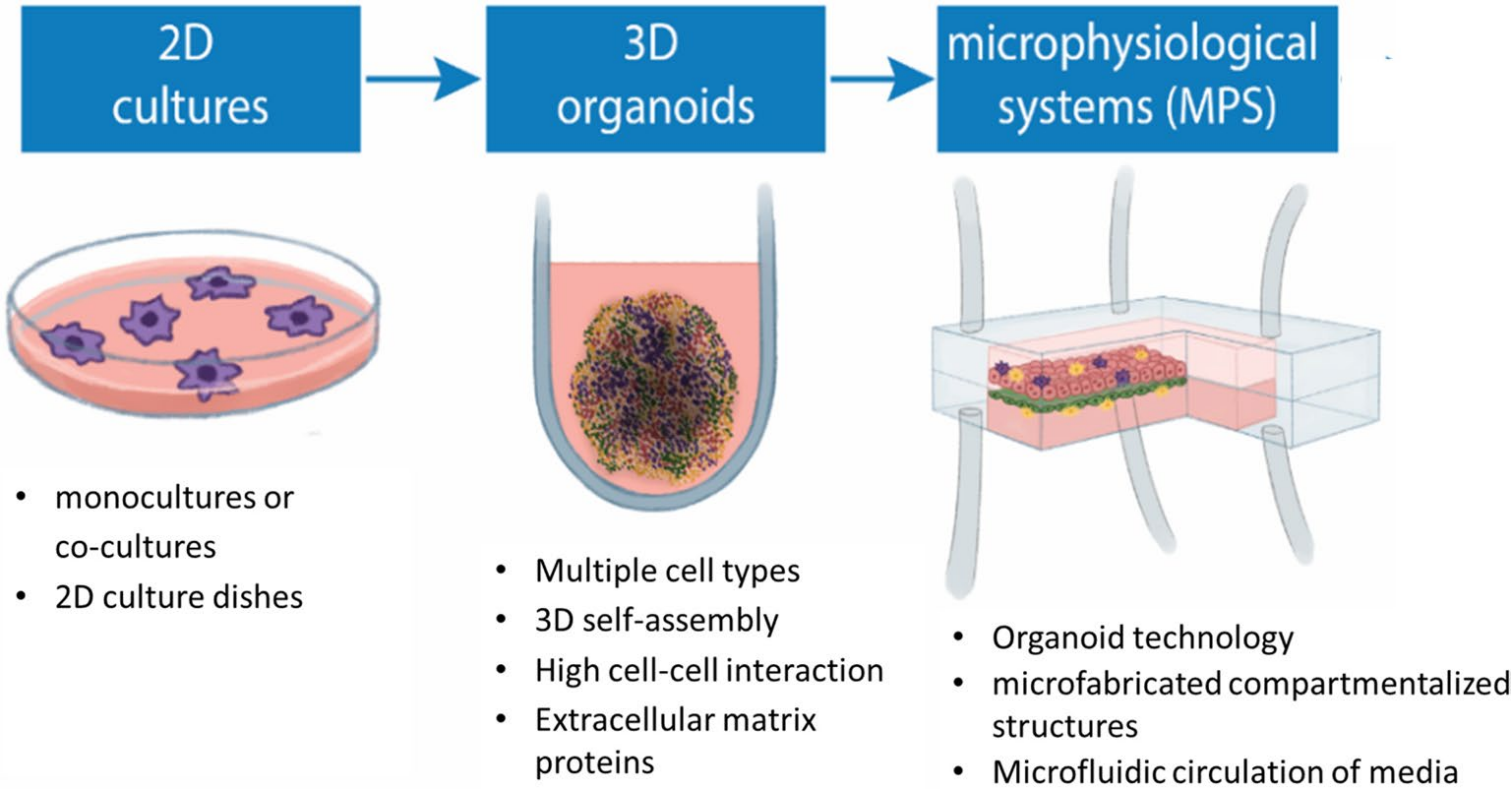
Evolution of in vitro culture systems from 2D to advanced microfluidic platforms. Traditional 2D monocultures or co‐cultures on flat dishes offer limited cell–cell interaction and lack tissue architecture. In contrast, 3D organoid systems enable the self‐assembly of multiple cell types with extracellular matrix support, better mimicking in vivo conditions. The most advanced models incorporate microfabricated compartments and microfluidic media flow to further enhance physiological relevance and control over the cellular microenvironment (adapted from [152]).

Several organoid approaches have been applied or adapted to study B cell responses. One of the earliest models was the Human Artificial Lymph Node (HuALN), introduced by Giese et al. in 2006. This bioreactor‐based system combined a 3D hydrogel culture with PBMCs, DCs, and a viral antigen, leading to the self‐assembly of lymphoid‐like structures and PC formation [151]. While it demonstrated the potential of immune organoids, it lacked class switching and affinity maturation. More recent organoid models have become more complex (Figure 4). Tonsil‐derived organoids, for example, leverage the lymphoid nature of tonsillar tissue and support key GC activities in vitro. When cultured in 3D matrices with appropriate stimuli, these organoids maintain a diverse immune cell repertoire and exhibit class switching, somatic hypermutation, and antigen‐specific antibody production [153, 154, 155]. Beyond traditional static organoid models, microphysiological systems (MPS) offer a complementary approach to increase physiological relevance. By incorporating microfluidics, perfusion, and engineered scaffolds, MPS better mimic vascularization, cytokine gradients, and tissue–tissue interfaces, supporting immune cell viability and dynamic interactions. Combining MPS with lymphoid organoids could extend GC‐like activity. One example for dynamic microphysiological organoid models is an organ‐on‐a‐chip model with vascular‐like perfusion systems, which can help recapture the critical role of blood and lymphatic flow [155]. PBMC‐derived immune organoids offer a more accessible alternative using peripheral blood from donors. In the presence of defined cytokines (e.g., IL‐4, IL‐21) and antigens, these cultures support B cell proliferation and differentiation into plasmablasts and MBCs and antigen‐specific antibody secretion [156, 157]. However, while they reflect key functional immune responses, they do not fully replicate the cellular origin of native lymphoid organs, highlighting a trade‐off between physiological relevance and practical feasibility.

In a recent study, organoid systems were further advanced by incorporating fibroblastic reticular cells (FRCs)– key stromal components essential for lymph node architecture and immune function [158]. Integration of ex vivo–cultured autologous FRCs into a lymph node‐derived hydrogel model improved overall immune cell viability, especially of B cells. This model also enhanced the secretion of immune‐regulatory cytokines like BAFF, CXCL12, CCL19, and IL‐6, offering a more physiologically relevant platform to study human immune responses in vitro [158]. However, while these diverse organoid models demonstrate significant potential for studying B cell responses, all reported findings have focused on cytokine or vaccine stimulation, whereas the potential of allergen‐specific activation remains poorly explored. To date, most studies on allergic sensitization have relied on 2D culture systems and murine models [159, 160]. MALT organoids hold a promising solution for allergy research, particularly through the integration of epithelial cells, a component that has been underutilized to date. Including epithelial–immune cell interactions is a crucial next step, as their crosstalk governs barrier function and cytokine signaling, thereby enabling more physiologically relevant models of allergic sensitization [16, 17].

Conventional animal models, especially humanized mouse models, and 2D cultures have been invaluable for understanding how the immune system works, but they also have significant limitations. One of the major challenges is that humanized mouse models generate a hybrid immune environment, in which engrafted human immune cells coexist and interact with murine components, rather than recapitulating a complete human immune architecture. This mismatch affects the authenticity of immune cell interactions and disease development [161, 162, 163, 164, 165].

Although organoid models of lymphoid tissues can overcome some of these challenges, limitations remain. The duration and intensity of GC‐like activity may be limited, and reproducibility across donors or laboratories may vary. In addition, fibroblast integration into organoids is technically challenging and requires careful optimization to maintain the viability and function of both compartments over time. It remains to be seen how modeling allergen‐specific IgE responses can be achieved in organoids despite the natural rarity of IgE+ B cells [152].

## 8 Conclusions and Outlook

The nasal mucosa is a critical site for interaction with allergens as it is both a barrier and an immunologically active tissue. Changes in epithelial cells, especially basal and goblet cells, and shifts in the nasal microbiome contribute to barrier dysfunction and allergic inflammation. Allergen source‐derived proteases and environmental factors further compromise the integrity of the epithelium and enhance sensitization. Future research should focus on clarifying the contribution of the microbiome, identifying the molecular drivers of epithelial remodeling, and developing measures to restore barrier function. New transcriptomic insights and organoid models could enable personalized therapies targeting early immune activation and epithelial resilience in allergic airway diseases.

Allergens enter the lymph nodes through passive lymph flow or active transport by DCs. Through passive transport via the lymph, low‐molecular‐weight allergens reach the B‐cell follicles via the conduits, while high‐molecular‐weight allergens are transported by macrophages. Migratory cDC2 actively transport allergens and prime TFH cells and IgE responses. Future strategies could target these transport pathways to influence allergic sensitization and improve immunotherapy.

The activation of naïve B cells and the formation of IgE PCs are central processes that trigger allergic sensitization and persistent IgE reactions. Recent findings show that naïve B cells undergo several activation pathways—either directly via IgD + −naïve cells or via IgG1 intermediates—that lead to rapid differentiation into IgE PCs. These cells often bypass the prolonged GC reactions and lead to short‐lived but highly pathogenic IgE responses. New therapeutic strategies, including targeted modulation of naïve B cell activation, induction of regulatory B cells, and enhancement of IgE‐blocking antibodies, promise to shift the immune response from hypersensitivity to tolerance. Future studies should explore long‐lived IgE PC reservoirs, optimize monoclonal IgG blocking strategies, and develop early interventions to block B‐cell activation prior to the formation of a pathological IgE response.

Organoid models provide a powerful platform for studying B cell activation by mimicking a complex immune microenvironment and overcoming the limitations of 2D cultures and mouse models. Future efforts should focus on integrating epithelial and stromal components, improving reproducibility, and developing allergen‐specific organoid systems to advance allergy research and precision immunotherapies (Box 2).

BOX 2 **Open research questions and future aims of the field.**

- How do the various environmental factors that cause epithelial barrier defects specifically induce B cell activation and class switching in allergic diseases?
- What mechanisms regulate the contribution of nasal IgE production to the systemic IgE reservoir?
- What molecular mechanisms are involved in cDC2‐dependent induction of IgE‐promoting T follicular helper cells? Could blockade of CD28/CTLA4 and CD80/CD86 costimulation be used to interfere with B‐cell help?
- Can anti‐IL‐33 monoclonal antibodies be used to inhibit extrafollicular IgE switching induced by ILC2s?
- Which routes of allergen delivery to the LN are relevant for restimulation of B cells?
- What mechanisms lead to the escape of highly mutated allergen‐specific IgE clonotypes found within the GC?
- How can organoid models be optimized to support allergen‐specific B cell activation and sensitization processes?
- How do aging‐related changes in B cells contribute to the development and progression of allergies?

## Author Contributions

O.G. and H.B. conceived the original idea and outline of this article. All authors contributed equally to the design, writing, reviewing, and editing of the manuscript and have approved its final version.

## Funding

This work was supported by the Austrian Science Fund (FWF) grants 10.55776/PAT3959823 (OG), P32953 and I4437 (BB), P36664 (KMS), 10.55776/KLP4891723 (JED), by the Federal State of Lower Austria Danube‐ARC P03 (BB), P04 (HB), P10 (TK), by the Deutsche Forschungsgemeinschaft grants EXC 22167‐390884018 (RAM), MA 2273/16‐1 (CCU), and by the Junior Program of the Medical Section of the University of Lübeck: J06/2024 (CCU).

## Conflicts of Interest

J.E.‐D. served as a speaker and/or consultant and/or advisory board member for Sanofi, Allergopharma, AstraZeneca, GSK, and Novartis. J.E.‐D. is an investigator for Novartis and AstraZeneca grants paid to her institution. All other authors declare that they have no conflicts of interest.

## Data Availability

Data sharing not applicable to this article as no datasets were generated or analyzed during the current study.
